# Sexual dysfunction precedes motor defects, dopaminergic neuronal degeneration, and impaired dopamine metabolism: Insights from *Drosophila* model of Parkinson’s disease

**DOI:** 10.3389/fnins.2023.1143793

**Published:** 2023-03-21

**Authors:** Zevelou Koza, Mohamad Ayajuddin, Abhik Das, Rahul Chaurasia, Limamanen Phom, Sarat Chandra Yenisetti

**Affiliations:** Drosophila Neurobiology Laboratory, Department of Zoology, Nagaland University (Central), Lumami, Nagaland, India

**Keywords:** Parkinson’s disease, *Drosophila*, courtship behavior, dopamine, sexual dysfunction

## Abstract

Sexual dysfunction (SD) is one of the most common non-motor symptoms of Parkinson’s disease (PD) and remains the most neglected, under-reported, and under-recognized aspect of PD. Studies have shown that Dopamine (DA) in the hypothalamus plays a role in regulating sexual behavior. But the detailed mechanism of SD in PD is not known. *Drosophila melanogaster* shares several genes and signaling pathways with humans which makes it an ideal model for the study of a neurodegenerative disorder such as PD. Courtship behavior of *Drosophila* is one such behavior that is closely related to human sexual behavior and so plays an important role in understanding sexual behavior in diseased conditions as well. In the present study, a sporadic SD model of PD using *Drosophila* was developed and SD phenotype was observed based on abnormalities in courtship behavior markers. The *Drosophila* SD model was developed in such a way that at the window of neurotoxin paraquat (PQ) treatment [PQ is considered a crucial risk factor for PD due to its structural similarity with 1-methyl-4-phenyl pyridinium (MPP+), the active form of PD-inducing agent, 1-methyl-4-phenyl-1,2,3,6-tetrahydropyridine (MPTP)], it does not exhibit mobility defects but shows SD. The whole brain tyrosine hydroxylase immunostaining showed no observable dopaminergic (DAergic) degeneration (number of DA neurons and fluorescence intensity of fluorescently labeled secondary antibodies that target anti-TH primary antibody) of the SD model. Similarly, there was no significant depletion of brain DA and its metabolite levels (HVA and DOPAC) as determined using HPLC-ECD (High-Performance Liquid Chromatography using Electrochemical Detector). The present study illustrates that the traits associated with courtship and sexual activity provide sensitive markers at the earlier stage of PD onset. This PQ-induced SD fly model throws an opportunity to decipher the molecular basis of SD under PD conditions and to screen nutraceuticals/potential therapeutic molecules to rescue SD phenotype and further to DAergic neuroprotection.

## Introduction

Parkinson’s disease (PD) is the second most dominant neurodegenerative disorder that affects about 1% of the population over age 50 (Modi et al., 2016). Sexual dysfunction (SD) is one of the most common non-motor disorders affecting Parkinsonian patients and remains the most neglected, underreported, and underrecognized aspect of PD (Jitkritsadakul et al., 2015; Bhattacharyya and Rosa-Grilo, 2017; Vela-Desojo et al., 2020; Benigno et al., 2022; Bronner et al., 2022; Elshamy et al., 2022). The most frequently reported sexual malfunctions in PD men are a decline in libido/loss of sexual interest, decline in sexual intercourse, diminution in orgasm/inability to experience orgasm/orgasmic dissatisfaction, decline in erection, erectile dysfunction, diminution in ejaculation, and premature ejaculation (Koller et al., 1990; Wermuth and Stenager, 1995; Lucon et al., 2001; Sakakibara et al., 2001; Hobson et al., 2003; Jitkritsadakul et al., 2015; Bhattacharyya and Rosa-Grilo, 2017).

Neurotransmitter Dopamine (DA) which is highly conserved throughout evolution has been suggested to play an important role in normal sexual function (Hull et al., 2004; Andersson, 2011) and any disruption in the levels of DA leads to abnormal sexual behavior (Zahran et al., 2001; Shaltiel-Karyo et al., 2012). Studies have shown that DA in the hypothalamus plays a role in regulating sexual behaviors, however, the detailed mechanism of SD in PD is not known (Sakakibara et al., 2011). A study reported the relationship between the severity of SD and specific patterns of nigrostriatal dopaminergic (DAergic) denervation (especially involving both putamina) in newly diagnosed drug-naïve PD patients (Contaldi et al., 2022). The understanding of the function and regulation of the behavioral circuits involved in mating will have potential implications for the medical treatment of SD in humans (Emmons and Lipton, 2003).

*Drosophila melanogaster* has been used widely as a model organism to understand the pathophysiology of human disease (s) specifically neurodegenerative disorders (NDDs) like PD as several disease-causing genes and signaling pathways are conserved between fly and humans (Reiter et al., 2001; Muñoz-Soriano and Paricio, 2011; Varga et al., 2014; Rahul and Siddique, 2022; Suzuki et al., 2022). The shorter life cycle (10–12 days at 25^°^C), a large number of progenies, established genetic methods, and molecular biology tools to manipulate genome and generate mutants and further perform loss and gain of function analysis make *Drosophila* an effective model system in biomedical research (Ayajuddin et al., 2018). *Drosophila* models a network of behaviors that are closely connected to humans, such as courtship (Dickson, 2008) which is believed to be one of the finest behaviors displayed by the fruit fly, and grooming (Tauber et al., 2011). The courtship ritual of a normal fly consists of fixed action patterns which are followed by several discrete steps such as the orientation of the male toward the female, tracking or following of the female, approaching and tapping the female, enthralling the female with a species-specific love song, licking the female’s genitalia, attempt to copulate, rejection or acceptance of the male by the female, mounting and copulation by the male upon female’s acceptance, and disconnection of the genitalia followed *via* dismounting by the male (Spieth, 1974; Koza et al., 2021). Therefore, courtship behavior is very important in determining the sexual status of male and female flies.

The purpose of the present study is to characterize SD phenotype in the *Drosophila* model of PD, by taking advantage of fly courtship behavior markers and to understand if SD sets in before the onset of motor defects. As DA plays an important role in sexual function, levels of DAergic neurodegeneration in the fly brain were checked using whole-brain immunostaining through fluorescence microscopy with anti-tyrosine hydroxylase (TH) (a rate-limiting enzyme in the synthesis of DA) antibody labeling and quantification of brain-specific DA and its metabolites DOPAC (3,4-Dihydroxyphenyl acetic acid) and HVA (Homovanillic acid) levels were checked using High-Performance Liquid Chromatography with an Electrochemical Detector (HPLC-ECD). Here we characterized the courtship dysfunctions in a *Drosophila* model and showed that the male fly showed courtship abnormality although there were no visible motor defects, no observable DAergic neurodegeneration; no variation in the level of synthesis of DA, HVA, DOPAC, and their turnover rate. This model illustrates that SD precedes motor defects in PD, hence it is of great value in understanding the progression of PD before the DAergic degeneration sets in. Therefore, this model will further throw an opportunity to screen nutraceuticals/potential therapeutic molecules to assess their efficacy to rescue SD and for possible DAerigc neuroprotective efficiency.

## Materials and methods

### *Drosophila* stock and husbandry: A collection of virgin and bachelor flies

Oregon K (OK) flies of *Drosophila melanogaster* used in this study were obtained from the National Drosophila Stock Centre (Department of Biotechnology, India supported) of the University of Mysore, Mysuru, Karnataka, India. Flies were maintained under standard laboratory conditions of 22 ± 2^°^C with a 12:12 h light and dark cycle (*Drosophila* environmental chambers from Percival, USA). The adult flies were propagated in media containing sucrose, yeast, agar-agar, and propionic acid in a definite standardized proportion (Phom et al., 2014).

For collecting unmated male and female flies, the adult flies were cleaned off from the culture vials and the newly emerged flies were collected within 2 h of emergence. For collecting the flies, they were mildly anesthetized with a few drops of diethyl ether. Male and female flies were separated and aged in same-sex groups of 25 in each vial. The collected flies were transferred to a fresh media vial every other day till they reached the age of 5 days old.

### Chemicals

The required chemicals viz., Methyl viologen dichloride hydrate/Paraquat (PQ) (Sigma-Aldrich; St. Louis, MO, USA, Cat. No. 856177), Sucrose (SRL, Maharashtra, India, Cat. No. 1947139), DMSO (Sigma-Aldrich; St. Louis, MO, USA, Cat. No. D8418). Phosphate buffered Saline (PBS; HiMedia, Maharashtra, India, Cat. No. ML023), Trichloro Acetic Acid (TCA; SRL, Maharashtra, India, Cat. No. 204842), Dopamine (DA; Sigma-Aldrich, St. Louis, MO, USA, Cat. No. H8502); 3,4-Dihydroxyphenylacetic acid (DOPAC; Sigma-Aldrich, St. Louis, MO, USA, Cat. No. 11569); Homovanillic acid (HVA; Sigma-Aldrich, St. Louis, MO, USA, Cat. No. 69673) were used for quantifying DA and metabolites. Paraformaldehyde (Sigma-Aldrich, St. Louis, MO, USA, Cat. No. I58127), Triton X-100 (Sigma-Aldrich, St. Louis, MO, USA, Cat. No. T8787), Triton X-100 (Sigma, St. Louis, MO, USA), Normal goat serum (NGS, Vector labs, CA, USA), VECTASHIELD mounting medium (Vector Labs, CA, USA, Cat: H1000), Rabbit anti-Tyrosine hydroxylase (anti-TH) polyclonal primary antibody (Millipore, MA, USA, Cat: Ab152) and Goat anti-rabbit IgG H&L (TRITC labeled) polyclonal secondary antibody (Abcam, MA, USA, Cat: Ab6718) were used for immunostaining. Whatman filter paper no. 1 disk was used as a feeding medium in the experiment in a 30 × 100 mm glass vial.

### Fly treatment

Ten mM PQ solution (treated) was prepared in 5% sucrose and a volume of 275 μL of treated and 5% sucrose (control) was pipetted on a filter disk placed on a 30 × 100 mm glass vial. Unmated male flies (5–6 days old) were treated with freshly prepared 5% sucrose (control) and 10 mM PQ solution (treated) for 2/3/4/5/6 h. Unmated female flies (5–6 days old) were used to study the PQ-induced male SD.

### Negative geotaxis assay

The motor ability of the flies was assessed through a negative geotaxis assay as described by Botella et al. (2004) and Phom et al. (2021). In brief, a single male was aspirated out from the vial using an aspirator and put in a plastic tube 26 cm long and 1 cm in diameter. The fly was then gently tapped to the bottom of the tube to acclimatize for 2 min. After 2 min, the fly was tapped gently to the bottom, and the height it could climb in 12 s was noted. This was repeated thrice for each fly and a minimum of 15 flies were observed for each group.

### Immunostaining to visualize DAergic neurons in the whole fly brain

Immunostaining assay was performed following the protocol of Ayajuddin et al. (2022). In brief, 5 days old male OK flies were fixed in 4% paraformaldehyde (PFA) containing 0.5% TritonX-100, at room temperature for 2 h. The brains were then washed 5 times for 15 min duration each (75 min) at room temperature with phosphate-buffered saline (PBS) containing 0.1% Triton-X 100 (PBST). Blocking was done using 5% Normal Goat Serum (NGS) in 0.5% PBST for 2 h at room temperature. Then, the brains were incubated for 72 h at 4^°^C with primary antibody (anti-tyrosine hydroxylase anti-TH, 1:250 dilution). The excess primary antibody was removed by washing brains in PBST [5 times with 15 min duration each (75 min)]. Brains were then incubated with secondary antibody (TRITC labeled, 1:250 dilution) for 24 h under the dark condition at room temperature, followed by a thorough wash in PBST [5 times with 15 min duration each (75 min)]. Then the brains were embedded in VECTASHIELD^®^ mounting medium and proceeded for image acquisition on the same day. Mounting of the whole fly brain for fluorescence microscopy (Carl Zeiss, Axio Imager M2 with ZEN 2012 SP2 software, Germany) was done as described in Ayajuddin et al. (2022).

The quantification of anti-TH signals was also performed following the protocol described in Ayajuddin et al. (2022). In brief, prepared/stained brains (3–5 brains for each group) were viewed under a fluorescence microscope at a 40 × magnification. A Rhodamine filter was used for image scanning. For image acquisition at 40 ×, a red dot test [for visibility of neuron (s) and assessing the signal saturation] was done for all the brains. Z-stack programming with constant intervals was performed. For image processing, on the method column, image subset and maximum intensity projection (MIP) with X-Y Plane were created. From 3D images of Z- stack, PAL, PPL1, PPL2, PPM1/2, and PPM3 (PAL, protocerebral anterior lateral; PPL, protocerebral posterior lateral; PPM, protocerebral posterior medial) brain regions were selected. The images were enlarged to see clear neuritis and a line was drawn around the neuron using draw spline contour from graphics tools and the intensity sum was created in .xml format. The procedure was repeated for all the neurons in different clusters. Care was taken to select the fly brains with the same orientation.

### Quantification of DA and its metabolites (DOPAC and HVA) using HPLC-ECD

Brain-specific DA and its metabolites were quantified using HPLC-ECD (HPLC-Thermo Scientific, Dionex Ultimate 3000) following the protocol described by Ayajuddin et al. (2021). Briefly, post-exposure control and PQ-fed groups of flies were quickly frozen. Followed by the procedure with a sharp scalpel 15 fly heads were decapitated on ice to prevent the thawing of tissue and degradation of biomolecules. Head tissues were homogenized in chilled 300 μL of PBS. The homogenate is subjected to sonication (for 20 s with 5 s interval at 30 percent amplitude) followed by centrifugation at 4^°^C for 10 min at 6,000 rpm. The supernatant collected after centrifugation was mixed with 5% TCA in a ratio of 1:1. 50 μL of the supernatant was kept aside for protein quantification before mixing with TCA. A composite standard mix comprising standard DA, DOPAC, HIAA, 5-HT, and HVA was prepared in PBS, each having a final concentration of 300 ng/mL. The solvent was mixed with 5% TCA in a ratio of 1:1 and kept chilled to avoid catecholamine degradation. A total of 20 μL of composite standard and 50 μL of the sample were loaded onto HPLC for quantification. MCM 15 cm 4.6 mm, 5 m C–18 packed column (Thermo Scientific, Cat: 70–0340) was used as the stationary phase for the elution of the catecholamines, while MD-TM from Thermo Scientific (Cat: 701332) was utilized as the mobile phase. A range of -175 to +225 mV was used for the reduction and oxidation potentials inside the primary ECD, which contains two cells, to detect the catecholamines. To cut down on background noise, Omnicell’s third cell, which serves as the secondary ECD module, was tuned to +500 mV. It was decided to use a 5 Hz data collection rate. Chromeleon^®^7 from Thermo Scientific, USA, was used to analyze chromatograms. The retention time of a catecholamine was compared between standard and sample chromatograms. To accurately pinpoint the DA, DOPAC, and HVA peak in the sample, 10 μL of the composite standard was mixed and the sample was run again in HPLC. The peaks that spiked according to the detection sequence were identified as the monoamines of interest.

Quantification and normalization of peaks were done as described in Ayajuddin et al. (2021).

In brief, (1). The concentration of a catecholamine of interest is: C_Std_ (ng/mL), (2). The area of the catecholamine in the standard chromatogram is: A_Std_ and the injection volume of the standard solution is: I_Std_ (μL), (3). Similarly, the area of the catecholamine in the sample chromatogram is: A_Samp_ and the sample injection volume is I_Samp_ (μL), (4). The total protein concentration of the brain extract is: P_Samp_ (μg/μL).

Calculation:

1. Concentration of the standard catecholamine in I_Std_ (μl) injection volume: (C_Std_ X I_Std_)/1,000 = V1 (ng).
2. Concentration of catecholamines in brain tissue extract sample: (A_Samp_ X V1)/A_Std_ = V2 (ng).
3. Total protein in I_Samp_ (μL) injection volume: (P_Samp_ X I_Samp_) = V3 (μg).
4. Determining the catecholamine concentration in total injected protein and normalizing for 1 mg (1,000 μg) of protein = (V2 × 1,000)/V3 = V4 (ng).
5. Since injected brain tissue extract and standard solution contain 5% TCA in a 1:1 ratio, the actual catecholamine concentration in brain tissue extract is (V4/2) = V5 (ng) in 1 mg of total protein.

The actual concentration values of each catecholamine of each experimental group were normalized to their respective control. The relative values of all runs were used to present the data and analyze the significance of the trend.

### Courtship assay

For each observation of courtship behavior, a single unmated and un-etherized male and female was transferred into a mating chamber (4 × 4 cm glass cavity block with an inner circular diameter of 3.3 cm) with the help of a mouth aspirator and allowed to acclimatize for 1 min and if the pair did not mate within 15 min, they were recorded as not mated and replaced by a fresh pair. After each observation, the mating chamber was cleaned thoroughly with 70% alcohol and air-dried. Courtship assay was conducted in 25 successful matings. All the observations were made from 8:00 to 15:00 h.

Below are male courtship activities that were observed (Spieth, 1974; Hegde and Krishna, 1997; Shaltiel-Karyo et al., 2012; Koza et al., 2021).

• NON-SEXUAL ENCOUNTER (NSE): Male and female come to cross each other but there is no sexual encounter.
• COURTSHIP LATENCY (CL): Time between the introduction of male and female together into the mating chamber until the orientation of the male toward the female.
• MATING LATENCY (ML): Time between the introduction of male and female together into the mating chamber until initiation of copulation of each pair.
• ATTEMPTED COPULATION (AC): The number of times the male attempt to copulate or mount the female.
• VIBRATION: Movement of the wings involves expanding them laterally from the resting position and then rapidly moving up and down.
• SCISSORING: The male repeatedly and rapidly extends both wings horizontally outward and back to the resting position.
• LICKING: Male opens labellar lobes, extends proboscis, and licks female genitalia.
• CHASING: The male fly chases the female for a few seconds or minutes until he is able to mount on the female.
• TAPPING: Male lifts, straightens foreleg, and strikes downward against the female; almost invariably occurs at the start of courtship.
• CIRCLING: Male periodically circles around the female, facing her as he moves, often from rear to front and back, sometimes completely about the female.

Videography.

All courtship assays were performed in the courtship chamber. The courtship behavior was recorded using a Microsoft 1080 HD sensor and video editing was done with WondershareFilmora v8.3 software.

Link to video on courtship behavior:

drive.google.com/file/d/1EapWLpCdxeqtcTK3WMX9Iyw79mtoZt-p/view?usp=sharing.

### Statistical analysis

Graph Pad Prism 5 software was used for statistical analysis. Significant differences between the two groups were analyzed using a two-tailed Student’s *t*-test. For more than two groups, one-way ANOVA (with Tukey’s post-hoc correction for multiple comparisons) was used. The error bar represents the standard error of the mean (SEM). Sexual activity of PQ-treated flies was represented in percentage (%) with reference to control fly performance (PQ-treated/control × 100).

## Results

### Characterization of PQ treatment window at which fly does not exhibit motor dysfunctions

**FIGURE 1:**
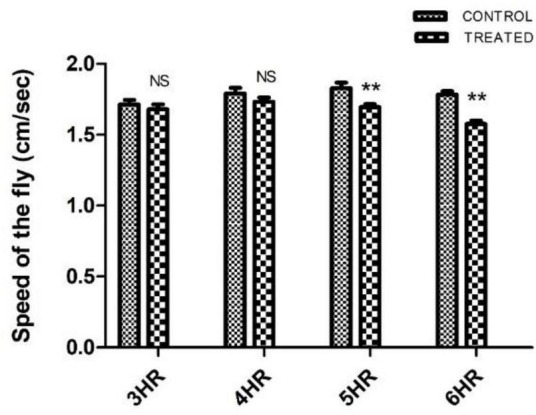
Characterization of paraquat (PQ) exposure window at which fly does not exhibit motor dysfunctions as determined by negative geotaxis assay: *Drosophila* male was exposed to 10 mM PQ and 5% sucrose (control) at different time points. A climbing assay was performed to check the motor ability of the fly. Fly started showing motor defects from 5 h of treatment onward. However, there was no significant difference in the motor ability between control and treated flies up to 4 h of treatment, providing an opportunity to understand SD well before the onset of mobility defects. (** signifies *P* < 0.01; NS: not significant).

Paraquat-induced motor dysfunction was assessed by exposing the male flies to PQ and subjecting them to a negative geotaxis assay. Male flies of age 5–6 days (the age of flies tested in the courtship assay) were exposed to PQ at different time points and were assessed to check their mobility efficiency. Idea is to select a time point of PQ exposure where the fly does not exhibit any mobility defects. Flies showed no difference in climbing speed at 4 h of PQ exposure as compared to the control flies which were on 5% sucrose for 4 h. Further exposure i.e., 5 h onward, the fly shows climbing defects as compared to the control (Figure 1). Thus, our observation at 4 h of PQ exposure where there were no mobility defects (compared to the control fly) provides an opportunity to screen for SD, if any, and will help further to characterize abnormalities in courtship behavior before the onset of mobility defects.

### Whole brain immunostaining illustrates no difference in DA neuronal number and TH synthesis levels in PQ-induced SD fly

**FIGURE 2:**
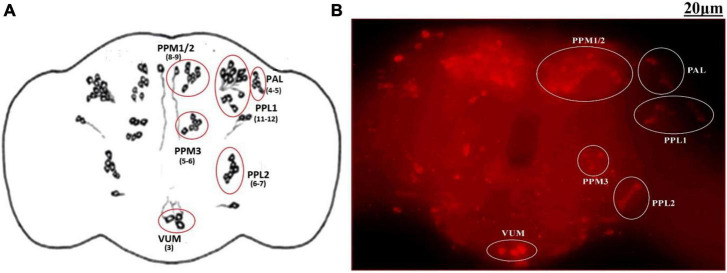
Quantifiable dopaminergic neuronal clusters in whole brain of *Drosophila*: cartoon of *Drosophila melanogaster* brain illustrating the position of quantifiable DAergic neurons **(A)** and image of the whole-brain mount of 5 days old male *Drosophila* captured by *ZEN software* of Carl Zeiss Fluorescence Microscope using fluorescently labeled secondary antibody targeted against the primary anti-TH antibody **(B)**. There are around 141 dopaminergic neurons (including ∼100 neurons of the PAM cluster which cannot be quantified) arranged in different clusters in each hemisphere. (PAL, proto-cerebral anterior lateral; PAM, proto-cerebral anterior medial; PPL, proto-cerebral posterior lateral; PPM, proto-cerebral posterior medial; VUM, ventral unpaired medial).

**FIGURE 3:**
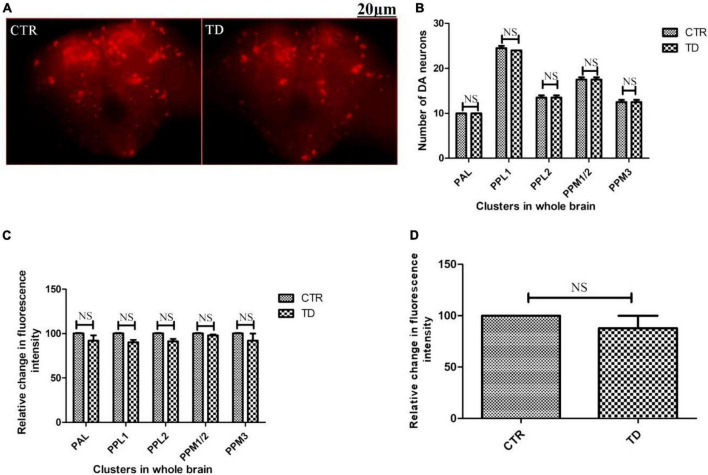
Characterization of DAergic neurodegeneration in the whole fly brain of control and paraquat (PQ)-induced sexual dysfunction (SD) fly **(A)** through anti-TH antibody immunostaining reveals that there is no loss in the number of DA neurons *per se* **(B)** and no change in the level of TH synthesis in all the clusters **(C)**, and *in toto* **(D)** between the control and treated group as analyzed by quantification of fluorescence intensity of fluorescently labeled secondary antibody that targets the anti-tyrosine hydroxylase (TH) primary antibody. (CTR, control; TD, treated with 10 mM PQ; Represented images are “merged” Z-stacking images; however, the quantification of DA neuronal number and fluorescence intensity is performed in 3D Z-stack images; PAL, protocerebral anterior lateral; PPL, protocerebral posterior lateral; PPM, protocerebral posterior medial). The scale bar of the brain images in the panel is 20 μm. Statistical analysis was performed using a *t*-test (compared to the control, NS, not-significant).

The adult *Drosophila* brain comprises six quantifiable DA neuronal clusters in each brain hemisphere (Figures 2A, B). To understand the DA neuronal dysfunction in the whole brain, 5 days old male fly model was immuno-stained for TH (the rate-limiting enzyme in the synthesis of dopamine) (Figure 3A). Upon visualizing the number of DA neurons, no significant difference was observed in all the clusters analyzed between the control and PQ-induced SD fly group (Figure 3B). Subsequently, to decipher if there is any change in the level of synthesis of TH, we quantified the fluorescence intensity of the DA neurons (fluorescently labeled secondary antibody targets the primary anti-TH antibody, and hence fluorescence intensity is correlated to the level of TH protein synthesis). Upon quantifying the fluorescence intensity of DA neurons, results illustrate that there is no difference in the fluorescence intensity in all the neuronal clusters (Figure 3C) and *in toto* (Figure 3D) between the control and the PQ treatment group. This confirms that there is no change in the levels of the rate-limiting enzyme of DA synthesis.

### HPLC-ECD data revealed that there is no difference either in DA or in its metabolites in the PQ-induced SD fly model

**FIGURE 4:**
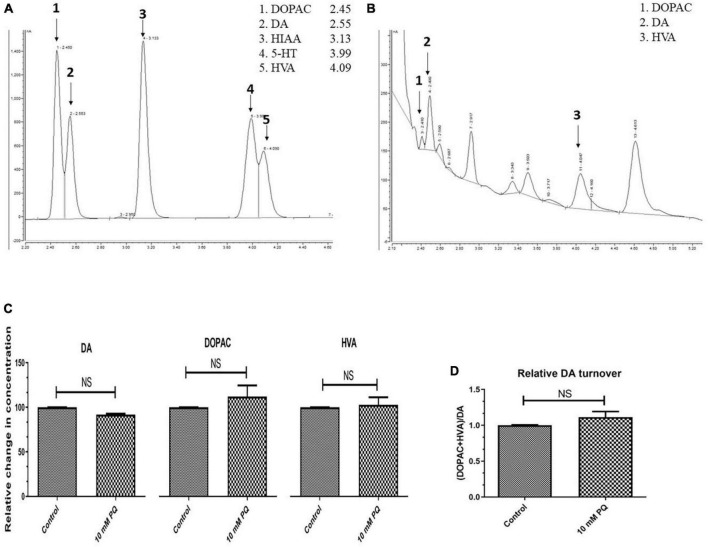
Quantification Dopamine (DA) and its metabolites- 3,4-Dihydroxyphenyl acetic acid (DOPAC) and Homovanillic acid (HVA) using High-Performance Liquid Chromatography (HPLC) in fly brain homogenate: The retention time of standard DA, DOPAC, and HVA is shown in the chromatogram **(A)** and chromatogram for the fly brain homogenate shows the detected monoamines **(B)**. The relative level of DA and its metabolites (DOPAC and HVA) shows that there is no significant difference between the control and PQ-treated groups **(C)**. Results also revealed that in PQ treated group compared to the control, there is no alteration of DA catabolism to DOPAC and HVA, as represented by unaltered relative DA turnover **(D)**. Statistical analysis was performed using a *t*-test (compared to the control), NS, not-significant.

Further to confirm whether there is DA synthesis depletion in PQ-induced SD fly, quantification of brain-specific DA and its metabolites was performed in tissue extract from the fly heads using the HPLC-ECD method (Figures 4A, B). The result shows that the SD model does not lead to any significant depletion of the DA level (Figure 4C). Further analysis revealed that there is no difference in the levels of DA metabolites i.e., DOPAC and HVA (Figure 4C). Further, there is no difference in the DA turnover which signifies no alteration in the DA catabolism (Figure 4D).

### Characterization of sexual behavior illustrates courtship behavior anomalies before the onset of motor defects

**FIGURE 5:**
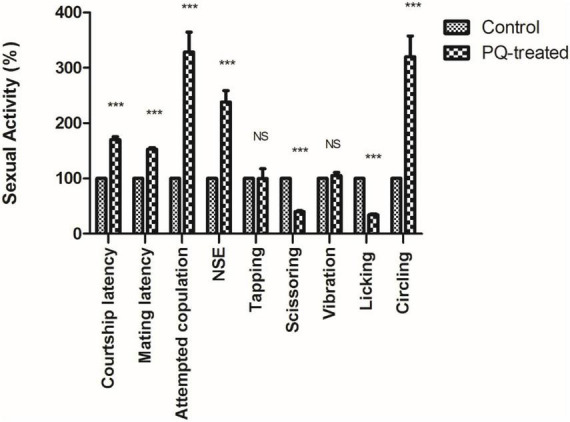
Sexual dysfunction precedes motor dysfunctions in the *Drosophila* model of Parkinson’s disease (PD). Paraquat (PQ) treated males exhibited following courtship anomalies when compared to control males, such as increased courtship latency, mating latency, attempted copulation, NSEs, and circling behavior but decreased scissoring and licking behavior. However, there was no difference between control and treated in behaviors such as tapping and vibration. A Statistical analysis was performed using a *t*-test (compared to control). ***p < 0.0001. NS, not-significant.

**FIGURE 6:**
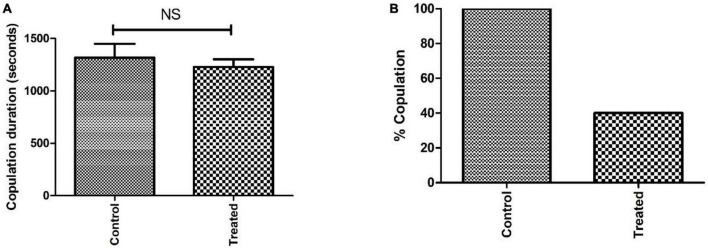
Analysis of sexual behavior before the onset of mobility defects showed that the difference in copulation duration between control and treatment is not significant (NS) **(A)**. However, the difference in percent copulation is about 60% less in male-treated mating (20 males mated successfully/25 in the control group and 9/25 in the treated group) when compared to control mating **(B)**.

The courtship activity was normalized to consider control male flies as ideal (100% sexual activity). Males with PQ treatment show significant courtship disparity when compared to control males (Figure 5). Courtship markers like attempted copulation, NSEs, and circling were increased by 200, 140, and 200%, respectively, in PQ treatment males as compared to the control males. Courtship latency and mating latency were increased by 70 and 60%,respectively, as compared to the control group; however, courtship behaviors like scissoring and licking were decreased by 60% in PQ-treatment males as compared to control. There was no difference between control and treated males in courtship activities like tapping and vibration (Figure 5). The duration of copulation between the males and the females showed no difference in control and treated males, but there was a significant difference in the percentage of successful copulation as treated males showed 60% less in successful copulation compared to their control counterparts (Figure 6).

## Discussion

*Drosophila* has been widely used as a model animal to study NDDs like PD as they share the same types of neurotransmitter systems like gamma-aminobutyric acid (GABA), glutamate, DA, serotonin, and acetylcholine, and can perform complex behaviors viz., sexual display, social behavior, and learning (Botella et al., 2009). SD in PD seemed to be multifactorial with no single cause identified (Brown et al., 1990) but the involvement of DA remains one of the most important factors (Heaton, 2000; Contaldi et al., 2022). However, very little is known about the SD mechanisms involved in sporadic PD. And very few studies have been done to understand the effects of PQ that led to neurodegenerative disorders such as PD and their effects on sexual functions.

In the present study, we report courtship/sexual behavioral deficits in the male fly even before there were any mobility defects. Epidemiological studies have also shown that most PD patients had experienced SD before being diagnosed with PD (Bronner et al., 2022), A decline in climbing ability is a convenient behavioral measure of degeneration of DAergic neurons/neurological damage in *Drosophila* (Feany and Bender, 2000). Previously our laboratory demonstrated that exposure to young flies (5–6 days old) with 10 mM PQ for 24 h shows a 30% decline in climbing speed, imitating PD-like symptoms of resting tremor, bradykinesia, and depleted brain DA levels (Phom et al., 2014). In human PD patients, tremors and other motor signs are diagnosed only when 50–60% of DAergic neurons degenerate leading to a 70–80% depletion of DA levels in the dorsal striatum where these neurons project (Fearnley and Lees, 1991). This is the prime reason to study courtship dysfunction (s) before the onset of motor dysfunction. We examined eleven components of male courtship behavior and in eight traits the PQ-treated flies exhibit defective behavior, suggesting PQ treatment though did not cause mobility defects in male flies, it induced courtship dysfunctions.

In the present study of the sporadic PD model, male exhibits the following courtship disparity when compared to control males, (1) decreased scissoring behavior (60%), (2) decreased licking behavior (60%) besides a significant decrease in the percentage of successful copulation (60%). But an increase/enhancement in the following behavior was observed: (1) CL (70%), (2) ML (60%), (3) AC (150%), (4) NSEs (140%), and (5) circling behavior (200%). However, there was no difference in behaviors such as tapping, vibration, and copulation duration between the control and treated group, which can be attributed to the differential genetic/molecular basis of regulation of different courtship markers. Chauhan et al. (2017) in their study using *D. melanogaster* as a model animal showed that after exposure to methylmercury (MeHg, a neurotoxic heavy metal), the male fly showed decreased wing-flapping behavior and failure to copulate with the female. The sporadic PD fly model showed an increase in CL after exposure to PQ. A similar observation of the increased CL was reported by Jantrapirom et al. (2020) in the ubiquilins (UBQLNs) depleted fly model. UBQLNs is an important group of proteins involved in proteostasis, which is also associated with pathological inclusions of Lewy bodies (intracytoplasmic proteinaceous inclusions) in the PD brain, the UBQLNs depleted flies show a reduction in DA and serotonin levels and when paired for courtship, they showed a longer CL of nearly double that of control pairs. An alteration of either copulation processes such as an extension of CL or a shortening of the copulation period might reveal some defects in courtship behaviors (Jantrapirom et al., 2020).

Shaltiel-Karyo et al. (2012) in their study using alpha-synuclein (α-syn) A30P mediated PD fly model reported impairment of courtship traits such as orientation, vibration, licking, AC, NSEs, and copulation when compared to control. Observed similarities of courtship behavioral markers between genetic and sporadic PD fly models, illustrate the involvement of DAergic pathways in the distorted courtship behavior.

It would be interesting to study further and figure out the interaction between genes and the environment in PD, for which the present model would be of immense help. It will further help to screen potential therapeutic molecules for PD.

Feany and Bender (2000) first reported the *Drosophila* model of PD by expressing normal and mutant forms of α-syn and showed the adult-onset loss of DAergic neurons. Several studies in PD models have shown the varying level of DAergic cell loss in various brain DA clusters (Auluck et al., 2002; Chen and Feany, 2005; Cooper et al., 2006; Trinh et al., 2008, 2010; Barone et al., 2011). However, in the present study, the SD model does not exhibit variation in DA neuronal number i.e., there was no observable DA degeneration (both in the number of DA neurons and fluorescence intensity of fluorescently labeled secondary antibodies that target anti-TH primary antibody) in the brain. There was also no variation in DA and its metabolite levels (DOPAC and HVA) in the PQ-induced SD model compared to the control. Further, the result demonstrated no ques that would lead to the postulate that there is enhanced DA degradation in the PQ-induced SD model compared to the control. Loss of DA neurons in the fly models of PD has been an issue of controversy. Navarro et al. (2014) in their study using three different flies PD model systems, viz. genetic (α-syn, Pink1, parkin) and two toxins based (rotenone and PQ) models of PD also reported the absence of DAergic neuronal loss in all models tested. The possible explanation for the observation (absence of variation in the levels of DA and its metabolites) in this PQ-induced SD fly, could be due to the following reasons:

a. At the selected window of PQ exposure, there may be no alteration in the levels of TH, DA, DOPAC, and HVA.
b. There lies a possibility for cell type-specific variation of DA and its metabolites (in the present study quantification is performed in whole brain tissue).
c. If the minute variation were to exist in a cell type-specific fashion that may not be possible to detect using the present method due to the limitation of sensitivity levels.
d. Further, other than catecholamines, genes like *fruitless* (Ryner et al., 1996) and certain neuroendocrine secretions such as juvenile hormone (JH) regulate male courtship behavior (Zhang et al., 2021). It will be interesting to probe further the biological regulation of *fruitless* and JH under induced PD condition.

## Conclusion

The present study demonstrates that exposure to the neurotoxicant PQ leads to SD as characterized by male fly courtship behavior, which precedes motor defects and brain DAergic neurodegeneration and alteration in DA metabolism. Therefore, traits associated with courtship and sexual activity will help as sensitive early-stage markers to identify the later-onset of PD in the *Drosophila* model. As by the time motor defects set in, a significant amount of brain DAergic neurodegeneration already occurred, the present model will provide an opportunity to understand the progression of the incipient pathophysiology of PD. Further, this model will support the development of biological markers for PD. Further, it will be interesting to investigate whether the courtship dysfunction (s) will have any influence on the reproductive fitness of the parents, further on the development of the progeny. By taking advantage of the power of fly genetics, it will be possible to decipher the genetic basis of SD, knowledge of which may contribute to developing therapeutic strategies for PD in humans and to identify interacting partners of disease-causing genes; understanding which is critical to developing and screen novel therapeutic molecules for late-onset NDD such as PD which has few therapeutic options.

## Data availability statement

The original contributions presented in this study are included in the article/Supplementary material, further inquiries can be directed to the corresponding author.

## Author contributions

ZK performed the experiments, data acquisition, and analysis of the data. MA participated in the experiments relating to HPLC and fluorescence microscopy. AD participated in the experiments relating to brain dopamine metabolism and analysis of the data. RC participated in the experiments relating to whole brain immunostaining and analysis of the data. LP participated in the experiments relating to developing PQ-induced SD fly. ZK, AD, and RC drafted the manuscript. SY contributed to the conception and design of the study, interpreted the data, revised the manuscript, obtained funding, and supervised the study. All authors contributed to the article and approved the submitted version.
