# Supplementary material for: Sexual dysfunction precedes motor defects, dopaminergic neuronal degeneration, and impaired dopamine metabolism: Insights from Drosophila model of Parkinson’s disease
Source: Front Neurosci. 2023 Mar 21;17:1143793. doi: 10.3389/fnins.2023.1143793 (PMC10072259; doi:10.3389/fnins.2023.1143793)
Supplement: Supplementary file 1 [file Data_Sheet_1.PDF]

Calculation of concentration of catecholamines in fly brain:

(15 fly heads/group were used in the assay)

i. The concentration of the standard catecholamines: DA (DA_{Std}), DOPAC ($DOPAC_{Std}$) and HVA (HVA_{Std}) used in the HPLC assay was 300 ng/mL each.

ii. Injection volume of all standard catecholamines to the HPLC column was I_{Std} = 20 µL.

iii. Areas of the peak of the catecholamines (DA, DOPAC and HVA) in the standard chromatogram were

A_{DA_Std} = 94, A_{DOPAC_Std} = 86.22 and A_{HVA_Std} = 117.32

iv. Injection volume of tissue extract to the column was I_{Samp} = 50 µL.

v. Areas of the peak of catecholamines (DA, DOPAC and HVA) in the "Control" brain tissue sample chromatogram were A_{DA_Samp} = 4.24, A_{DOPAC_Samp} = 0.53 and A_{HVA_Samp} = 14.78.

vi. The brain tissue extract from the "Control" group that was used for HPLC assay, was quantified beforehand for total protein which was TP_{Samp} = 0.139 µg/ µL.

vii. The following steps were followed for calculating the actual amount of the catecholamines in tissue extract (**Table 1**).

Calculation:

Calculation Steps	Metabolites		
	DA	DOPAC	HVA
Step I: Concentration of standard catecholamines in 20 µl of injection volume	DA_{Std} X I_{Std}/1000<br>i.e. (300 X 20)/1000 = 6 ng	$DOPAC_{Std}$ X I_{Std}/1000<br>i.e. (300 X 20)/1000 = 6 ng	HVA_{Std} X I_{Std}/1000<br>i.e. (300 X 20)/1000 = 6 ng
Step II: Concentration of catecholamines in brain tissue extract	(A_{DA_Samp} X 6)/ A_{DA_Std}<br>i.e. (4.24 X 6)/94 = 0.27063 ng	(A_{DOPAC_Samp} X 6)/ A_{DOPAC_Std}<br>i.e. (0.53 X 6)/86.22 = 0.03688 ng	(A_{HVA_Samp} X 6)/ A_{HVA_Std}<br>i.e. (14.78 X 6)/117.32 = 0.75588 ng
Step III: Determining the total protein in 50 µl that was injected into the column	(TP_{Samp} X I_{Samp})<br>i.e. (50 X 0.139) = 6.95 µg	(TP_{Samp} X I_{Samp})<br>i.e. (50 X 0.139) = 6.95 µg	(TP_{Samp} X I_{Samp})<br>i.e. (50 X 0.139) = 6.95 µg
Step IV: Determining the catecholamine in 1 µg of total protein	0.27063/6.95 = 0.03894 ng	0.03688/6.95 =0.0053 ng	0.75588/6.95 = 0.10876 ng

Step V: Determining the actual amount of catecholamine as injected brain tissue extract and the standard solution had TCA in a 1:1 ratio	0.03894/2 = 0.01947 ng in 1 µg of total protein	0.0053/2 = 0.00265 ng in 1 µg of total protein	0.10876/2 = 0.05438 ng in 1 µg of total protein
Step VI: Determining the actual amount of catecholamine in each fly brain	(0.01947 X 1000)/15 = 1.30 pg	(0.00265 X 1000)/15 = 0.18 pg	(0.05438 X 1000)/15 = 3.63 pg

Table 1: Steps for calculation of the amount of catecholamines in 1 mg of total protein of tissue sample.

Raw data

**Raw values (pg/brain)**						**Relative Values**				
		Control	10 mM PQ					Control	10 mM PQ	
DA	Run 1	1.30	1.20			DA	Run 1	100	92.30	
	Run 2	0.80	0.72				Run 2	100	90.24	
	Run 3	1.21	1.13				Run 3	100	93.39	
DOPAC	Run 1	0.18	0.24			DOPAC	Run 1	100	135.82	
	Run 2	0.93	0.86				Run 2	100	93.31	
	Run 3	0.54	0.58				Run 3	100	107.41	
HVA	Run 1	3.63	3.16			HVA	Run 1	100	87.16	
	Run 2	4.45	5.16				Run 2	100	115.96	
	Run 3	4.54	4.80				Run 3	100	105.73	
	Turnover ratio						Turnover ratio			
			Control	10 mM PQ					Control	10 mM PQ
(DOPAC+HVA)/DA		Run 1	2.93	2.84		(DOPAC+HVA)/DA		Run 1	1.00	0.97
		Run 2	6.75	8.38				Run 2	1.00	1.24
		Run 3	4.20	4.76				Run 3	1.00	1.13
